# Contribution of Major Lifestyle Risk Factors for Incident Heart Failure in Older Adults: The Cardiovascular Health Study

**DOI:** 10.1016/j.jchf.2015.02.009

**Published:** 2015-07

**Authors:** Liana C. Del Gobbo, Shadi Kalantarian, Fumiaki Imamura, Rozenn Lemaitre, David S. Siscovick, Bruce M. Psaty, Dariush Mozaffarian

**Affiliations:** ∗ Department of Nutrition, Harvard School of Public Health, Boston, Massachusetts; † Department of Epidemiology, Harvard School of Public Health, Boston, Massachusetts; ‡ Medical Research Council Epidemiology Unit, Institute of Metabolic Science, University of Cambridge School of Clinical Medicine, Cambridge Biomedical Campus, Cambridge, United Kingdom; § Department of Medicine, University of Washington, Seattle, Washington; ‖ Department of Epidemiology, University of Washington, Seattle, Washington; ¶ Group Health Research Institute, Group Health Cooperative, Seattle, Washington; # Department of Medicine, Brigham and Women’s Hospital and Harvard Medical School, Boston, Massachusetts

**Keywords:** diet, heart failure, lifestyle, nutrition, physical activity, sodium, AHA 2020, American Heart Association 2020 dietary goals score, AHEI, Alternative Healthy Eating Index, BMI, body mass index, CHD, coronary heart disease, CI, confidence interval, HF, heart failure, HR, hazard ratio

## Abstract

**Objectives:**

The goal of this study was to determine the relative contribution of major lifestyle factors on the development of heart failure (HF) in older adults.

**Background:**

HF incurs high morbidity, mortality, and health care costs among adults ≥65 years of age, which is the most rapidly growing segment of the U.S. population.

**Methods:**

We prospectively investigated separate and combined associations of lifestyle risk factors with incident HF (1,380 cases) over 21.5 years among 4,490 men and women in the Cardiovascular Health Study, which is a community-based cohort of older adults. Lifestyle factors included 4 dietary patterns (Alternative Healthy Eating Index, Dietary Approaches to Stop Hypertension, an American Heart Association 2020 dietary goals score, and a Biologic pattern, which was constructed using previous knowledge of cardiovascular disease dietary risk factors), 4 physical activity metrics (exercise intensity, walking pace, energy expended in leisure activity, and walking distance), alcohol intake, smoking, and obesity.

**Results:**

No dietary pattern was associated with developing HF (p > 0.05). Walking pace and leisure activity were associated with a 26% and 22% lower risk of HF, respectively (pace >3 mph vs. <2 mph; hazard ratio [HR]: 0.74; 95% confidence interval [CI]: 0.63 to 0.86; leisure activity ≥845 kcal/week vs. <845 kcal/week; HR: 0.78; 95% CI: 0.69 to 0.87). Modest alcohol intake, maintaining a body mass index <30 kg/m^2^, and not smoking were also independently associated with a lower risk of HF. Participants with ≥4 healthy lifestyle factors had a 45% (HR: 0.55; 95% CI: 0.42 to 0.74) lower risk of HF. Heterogeneity by age, sex, cardiovascular disease, hypertension medication use, and diabetes was not observed.

**Conclusions:**

Among older U.S. adults, physical activity, modest alcohol intake, avoiding obesity, and not smoking, but not dietary patterns, were associated with a lower risk of HF.

Heart failure (HF) is a growing public health problem with substantial morbidity and mortality (1). In 2010, direct and indirect U.S. health care costs were $39.2 billion (2). Incidence is highest in those older than age 65 years—the most rapidly growing segment of the U.S. population—and is a leading cause of hospitalizations (3). Despite treatment advances, long-term prognosis remains poor (4). Therefore, identifying and targeting modifiable factors for primary prevention of HF is crucial for decreasing incidence and disease burden.

Although lifetime risk of HF differed among population subgroups 5, 6, 7, the relative contribution of major lifestyle risk factors was unclear. During middle age, men who adhered to ≥4 healthy lifestyle habits (not smoking, regular exercise, maintaining normal weight, modest alcohol use, consuming breakfast cereals, and consuming fruits/vegetables) had a lower lifetime risk of HF (10%; 95% confidence interval [CI]: 8% to 12%) than men who did not follow any of these lifestyle factors (21%; 95% CI: 17% to 26%) (7). However, more detailed dietary information was not reported, and generalizability might be limited because this cohort included male, predominately white physicians who were at a much lower HF risk than community-based populations 5, 6. In another cohort of Swedish men and women, adherence to the Dietary Approaches to Stop Hypertension (DASH) diet was associated with lower HF risk, but contributions of other lifestyle factors were not evaluated 8, 9. Thus, the relative importance of overall dietary habits and other lifestyle factors for development of HF remains uncertain.

To address this key public health question, we investigated the separate and combined impact of major lifestyle risk factors on HF in the Cardiovascular Health Study (CHS), a community-based prospective cohort.

## Methods

### Design and population

The CHS enrolled 5,201 ambulatory men and women age ≥65 years who were randomly selected from Medicare eligibility lists in 4 U.S. communities in 1989 to 1990 an additional 687 African-American participants were enrolled in 1992 (10). Baseline evaluation included standardized physical examination, diagnostic tests, and questionnaires to determine health status, medical history, and lifestyle risk factors. For this analysis, participants were excluded if they had prevalent HF or moderate and/or severe mitral or aortic regurgitation at baseline (n = 698), missing information on lifestyle risk factors, or implausible (<500 or >4,000 kcal/day) energy intake (n = 700). Participants with prevalent hypertension, diabetes, and coronary heart disease (CHD) were included to maintain generalizability; we also evaluated effect modification by these factors.

### Assessment of lifestyle

We evaluated dietary patterns, physical activity, alcohol use, smoking, and adiposity (body mass index [BMI] and waist circumference). Diet was assessed in 1989 to 1990 using a validated 99-item food frequency questionnaire (National Cancer Institute) (11), and again in 1995 to 1996 using a validated Willett food frequency questionnaire (12). Dietary patterns were evaluated as a time-varying exposure, with the cumulative average of intakes from the 2 food frequency questionnaires used to reduce within-person variation and obtain the best estimates of long-term dietary intake. Four dietary patterns were evaluated: Alternative Healthy Eating Index (AHEI), DASH, a score based on the American Heart Association 2020 dietary goals (AHA 2020) (13), and a Biologic pattern constructed based on a priori knowledge of CHD risk factors. Dietary components and scoring algorithms are presented in Online Table 1; scoring for the AHEI and DASH patterns has been described previously 14, 15, 16.

Walking habits, including average pace and distance, were assessed by self-report at baseline and annually at each follow-up, and leisure-time activity (modified Minnesota Leisure-Time Activities questionnaire) and exercise intensity (low, medium, or high) by self-report at the baseline, third, and seventh annual visits. Alcohol use and smoking status were assessed at each annual visit. Trained personnel used standardized methods to measure weight at each annual visit, and height and waist circumference at the baseline, third, and seventh annual visits. To minimize potential misclassification from measurement error and changes in lifestyle, repeated measures were used to update lifestyle exposures using time-varying covariates with cumulative averaging.

### Ascertainment of incident heart failure

Participants were followed by annual study clinic examinations with interim telephone contacts for 10 years and telephone every 6 months thereafter. Incident HF was adjudicated by a centralized events committee using outpatient and inpatient medical records, diagnostic tests, clinical consultations, and interviews. Confirmation of HF required: 1) diagnosis by a treating physician; 2) HF symptoms (shortness of breath, fatigue, orthopnea, or paroxysmal nocturnal dyspnea) plus signs (edema, rales, tachycardia, gallop rhythm, or displaced apical impulse) or supportive findings on echocardiography, contrast ventriculography, or chest radiography; and 3) medical therapy for HF, defined as diuretics plus either digitalis or a vasodilator. Because data on HF subtypes were incomplete for many participants, HF subtypes were not explored in this analysis.

### Statistical analysis

Cox proportional hazards were used to estimate hazard ratios (HRs) for quintile groups of each exposure, with time at risk until HF, death, or most recent follow-up date. The proportional hazards assumption was tested by evaluating the product of each exposure times the natural log time in the model. Missing covariate information on education (<1% missing) and income (6% missing) were imputed using data on age, sex, race, and enrollment site. To assess the independent effect of a given lifestyle factor, multivariate models were mutually adjusted for each of the other lifestyle factors, plus age, sex, race, enrollment site, education, and income. Lifestyle factors were evaluated in combination to estimate the proportion of cases in the population that might be attributable to suboptimal levels of these factors (population attributable risk). The population attributable risk was calculated as: p(RR – 1)/(1 + p[RR – 1]), where p is the prevalence of individuals not in the low-risk group and RR is the associated multivariable-adjusted relative risk. Upper and lower 95% CIs of the population attributable risk were derived using this formula as well as the upper and lower 95% CI estimates of the multivariable-adjusted relative risk.

Effect modification was evaluated in analyses stratified by age, sex, race, BMI, baseline drug-treated hypertension, diagnosed diabetes, and baseline CHD, with significance assessed using the Wald test, which was adjusted using the Bonferroni test for multiple comparisons. Main analyses were unadjusted for multiple comparisons. In the secondary analyses, individual dietary components of the Biological score and incident HF were evaluated. Sensitivity analyses restricted to those without prevalent CHD at baseline, and to participants with good, very good, or excellent self-reported health were conducted. A sensitivity analysis adjusting for baseline N-terminal pro–B-type natriuretic peptide was also evaluated. Analyses were conducted using Stata SE (version 12, StataCorp, College Station, Texas), with a 2-tailed α = 0.05.

## Results

At baseline, 61% of participants were women, and the mean age was 72 years. Most participants (89%) were Caucasians; approximately 11% were African American. Distributions of demographic and lifestyle factors at baseline are shown in Table 1. Adherence to most dietary pattern scores was modest, with mean scores ranging from 46 (AHEI) to 66 (AHA 2020) of a maximum 100 points. Across quintiles for all patterns, characteristics associated with higher scores (indicating healthier diets) included female sex, higher educational attainment, and higher income. Similarly, physical activity (leisure activity, walking pace), never or former smoking, and modest alcohol use were associated with higher scores. Baseline blood pressure and diagnosed diabetes showed inverse relations across quintiles of diet scores; prevalent hypertension medication use and CHD showed no consistent pattern across scores.

**Table 1:** Characteristics of Older Adults by Diet-Quality Scores (n = 4,490)∗

	Biologic		DASH		AHEI		AHA 2020	
	Quintile 1	Quintile 5	Quintile 1	Quintile 5	Quintile 1	Quintile 5	Quintile 1	Quintile 5
Diet score∗	21.1 ± 2.5	39.4 ± 2.3	16.7 ± 2.1	31.7 ± 1.6	21.4 ± 4.7	58.8 ± 5.3	33.6 ± 6.2	66.6 ± 2.9
Range	11–24	37–49	9–19	30–38	5.5–27.5	52.5–80.5	7–37	63–77
Standard score	42.2 (5.0)	78.8 (4.6)	41.8 (5.3)	79.3 (4.0)	24.7 (5.4)	67.0 (6.0)	42 (7.8)	83.3 (3.6)
Range	22–48	74–98	22.5–48	75–95	6.3–31.4	59.9–91.8	8.8–46.3	78.8–96.3
Age, yrs	72.5 ± 5.5	71.7 ± 4.9	72.1 ± 5.3	72.0 ± 5.1	72.7 ± 5.6	71.6 ± 4.7	72.4 ± 5.6	71.8 ± 4.9
Sex								
Male	58.3	23.7	59.2	33.6	56.3	29.6	55.8	33.4
Female	41.6	76.3	40.8	66.4	43.5	70.4	44.2	67.6
Race								
Caucasian	89.2	87.6	88.6	88.7	88.3	91.9	88.8	90.8
Non-Caucasian	10.8	12.3	11.4	10.9	11.7	8.1	11.2	9.2
Education								
<High school	40.2	16.8	31.3	25.2	46.2	11.0	37.5	20.5
High school	33.4	38.3	35.3	38.1	31.9	33.9	32.2	37.5
>High school	26.3	44.8	33.4	36.7	21.9	55.1	30.3	42.0
Income, U.S.$/yr†								
<25,000	70.3	52.8	63.4	59.1	74.9	42.5	70.4	53.3
25,000–49,999	22.0	30.1	23.2	28.4	19.1	35.0	20.6	29.6
≥50,000	7.6	17.1	13.4	12.5	6.0	22.6	9.0	17.1
Leisure activity, kcal/week	1,703 ± 2,116	2091 ± 2,097	1,755 ± 2,109	2,033 ± 1,987	1,772 ± 2,180	1,938 ± 1,920	1,746 ± 2,129	1,925 ± 1,924
Walking pace, mph								
<2	35.5	15.3	32.0	23.2	36.5	13.6	31.9	18.8
2–3	41.7	38.6	38.9	38.8	42.9	37.5	42.1	39.7
>3	22.8	46.0	29.2	38.0	20.6	48.9	26.0	41.6
Smoking								
Never	41.9	48.7	43.1	51.2	43.1	46.0	39.1	52.1
Former	41.8	43.2	45.0	38.9	39.8	46.7	44.1	38.5
Current	16.3	8.0	11.9	9.6	17.1	7.0	16.7	9.3
Alcohol use, drink/week								
0	49.6	38.8	44.4	44.0	63.1	23.1	48.2	40.5
<1	17.7	21.4	17.4	19.3	15.5	23.4	17.2	21.6
1–3	14.6	15.6	16.2	13.1	6.9	22.2	12.7	14.2
>3	18.0	24.2	22.0	23.6	14.4	31.3	21.9	23.8
BMI, kg/m^2^								
<22.0	4.8	4.0	3.4	4.5	5.3	4.4	5.8	3.8
22.0–24.9	34.2	35.3	34.0	34.4	33.3	39.9	34.7	32.5
25.0–29.9	42.7	42.6	42.8	40.3	40.1	43.2	42.2	44.3
≥30.0	18.3	18.1	20.8	20.8	21.3	13.5	18.3	20.4
Blood pressure, mm Hg								
SBP	139 ± 20	135 ± 19	139 ± 20	136 ± 18	140 ± 21	135 ± 19	139 ± 20	136 ± 19
DBP	72 ± 11	68 ± 13	72 ± 12	68 ± 12	72 ± 12	69 ± 12	72 ± 12	69 ± 11
Prevalent hypertension	41.7	40.9	40.7	42.0	42.8	38.1	39.2	42.8
CHD	15.8	16.7	15.3	16.7	14.9	16.3	16.4	16.9
Diabetes	21.9	17.9	21.9	18.3	23.5	14.1	23.4	18.8

Values are mean ± SD, range, or %. Dietary components of the Biologic pattern included: 1) fruits; 2) vegetables; 3) whole grains; 4) fish; 5) polyunsaturated to saturated fat ratio; 6) nuts/seeds; 7) red and processed meats; 8) sugar-sweetened beverages; 9) transfat; and 10) sodium. For DASH (Dietary Approaches to Stop Hypertension): 1) low-fat dairy; 2) fruits; 3) vegetables; 4) nuts and legumes; 5) whole grains; 6) red and processed meats; 7) sugar-sweetened beverages; 8) and sodium. For Alternative Healthy Eating Index (AHEI): 1) fruits; 2) vegetables; 3) nuts and soy protein; 4) cereal fiber; 5) polyunsaturated to saturated fat ratio; 6) transfat; 7) alcohol; 8) long-term multivitamin use; and 9) white:red meat ratio. For American Heart Association 2020 dietary goals score (AHA 2020): 1) fruits and vegetables; 2) fish; 3) fiber-rich whole grains; 4) nuts, legumes, and seeds; 5) sodium; 6) sugar-sweetened beverages; 7) processed meats; and 8) saturated fat. For scoring of dietary patterns, see Online Table 1.

BMI = body mass index; CHD = coronary heart disease; DBP = diastolic blood pressure; SBP = systolic blood pressure.

∗ Points obtained of a maximum score of 50 for the Biologic pattern, 40 for DASH, 87.5 for AHEI, and 80 for the AHA 2020 pattern. Standardized scores were scaled to a maximum score of 100 points.

† Missing values for income (6.3% missing) were imputed using data on age, sex, race, and enrollment site.

During 51,850 person-years (maximum follow-up: 21.5 years), 1,380 HF cases occurred. After adjustment for demographic and lifestyle variables, no dietary pattern was associated with incident HF (Table 2). Results were not materially different when energy-unadjusted patterns were analyzed or when dietary scores were evaluated continuously (data not shown). In contrast, physical activity measures (exercise intensity, walking pace, leisure activity, and walking distance) were each associated with lower HF incidence in demographic-adjusted multivariate models. When mutually adjusted for other lifestyle variables, including other physical activity metrics, the highest category of walking pace and leisure activity, but not exercise intensity and walking distance, were each independently associated with lower HF risk (Online Table 2).

**Table 2:** Hazard Ratios (95% CI) for Incident HF by Quintiles of Diet-Quality Scores in Older U.S. Adults (n = 4,490)

	Quintiles of Diet-Quality Scores					p Value for Trend∗
	1	2	3	4	5	
Biologic†						
Cases/person–yrs	254/9,702	310/9,903	208/10,539	257/10,632	218/11,073	
Multivariate	1.00 (ref)	1.21 (1.03–1.43)	1.08 (0.91–1.28)	1.12 (0.94–1.34)	1.04 (0.89–1.31)	0.96
+ Mediator adjusted	1.00 (ref)	1.18 (1.00–1.39)	1.05 (0.88–1.24)	1.08 (0.91–1.28)	0.99 (0.82–1.19)	0.62
DASH‡						
Cases/person–yrs	284/10,261	330/11,968	268/10,271	263/9,698	235/9,652	
Multivariate	1.00 (ref)	1.12 (0.96–1.32)	1.12 (0.95–1.33)	1.23 (1.03–1.26)	1.11 (0.93–1.33)	0.12
+ Mediator adjusted	1.00 (ref)	1.11 (0.95–1.31)	1.06 (0.90–1.26)	1.19 (1.00–1.41)	1.05 (0.88–1.26)	0.36
AHEI§						
Cases/person–yrs	301/9,655	290/9,779	303/11,274	258/10,270	228/11,445	
Multivariate	1.0 (ref)	1.06 (0.90–1.25)	1.04 (0.88–1.22)	1.04 (0.87–1.24)	0.94 (0.78–1.14)	0.51
+ Mediator adjusted	1.00 (ref)	1.01 (0.86–1.19)	1.01 (0.85–1.19)	1.00 (0.85–1.20)	0.90 (0.74–1.09)	0.33
AHA 2020‖						
Cases/person–yrs	281/9,766	283/10,569	309/10,368	267/11,100	240/10,618	
Multivariate#	1.00 (ref)	1.02 (0.86–1.21)	1.19 (1.01–1.40)	1.09 (0.92–1.30)	1.01 (0.84–1.21)	0.57
+ Mediator adjusted∗∗	1.00 (ref)	1.04 (0.88–1.22)	1.15 (0.97–1.35)	1.05 (0.89–1.25)	0.96 (0.80–1.15)	0.88

Values are hazard ratio (HR) (95% confidence intervals [CI]) based on cumulatively averaged a priori score, DASH score, the AHEI score, and the AHA 2020 score.

HF = heart failure; other abbreviations as in Table 1.

∗ Linear trend was tested by assigning the median value to participants in each quintile and entering this into the model as a continuous variable.

† Biologic dietary pattern comprised 10 components: 1) fruits; 2) vegetables; 3) whole grains; 4) fish; 5) polyunsaturated to saturated fat ratio; 6) nuts/seeds; 7) red and processed meats; 8) sugar-sweetened beverages; 9) transfat; and 10) sodium. For scoring of dietary patterns, see Online Table 1.

‡ DASH dietary pattern comprised 8 components: 1) low-fat dairy; 2) fruits; 3) vegetables; 4) nuts and legumes; 5) whole grains; 6) red and processed meats; 7) sugar-sweetened beverages; and 8) sodium.

§ AHEI dietary pattern comprised 9 components: 1) fruits; 2) vegetables; 3) nuts and soy protein; 4) cereal fiber; 5) polyunsaturated to saturated fat ratio; 6) transfat; 7) alcohol; 8) long-term multivitamin use; 9) white to red meat ratio.

‖ AHA 2020 dietary pattern comprised 8 components: 1) fruits and vegetables; 2) fish; 3) fiber-rich whole grains; 4) nuts, legumes, and seeds; 5) sodium; 6) sugar-sweetened beverages; 7) processed meats; and 8) saturated fat.

# Multivariate model: adjusted for age (years), sex (male vs. female), race (Caucasian vs. non-Caucasian), enrollment site (4 clinics), education (less than high school, high school, more than high school), annual income (<$25,000, $25,000 to $49,999, >$50,000), kilocalorie of physical activity (quintiles), walking pace (<2, 2 to 3, >3 mph), smoking (never, former, current), alcohol intake (0, <1, 1 to 2, ≥3 drinks/week).

∗∗ Mediator adjusted: Multivariate model + additional adjustment for potential mediators, including body mass index (kilograms divided by square meters), prevalent treated hypertension (yes vs. no), prevalent diabetes mellitus (yes vs. no), prevalent coronary heart disease (yes vs. no). Additional adjustment for other potential mediators, such as fasting glucose, fasting insulin, blood pressure, triglycerides, or C-reactive protein to the mediator-adjusted model had no influence on model estimates and were not included in mediator-adjusted models.

**Figure 1:**
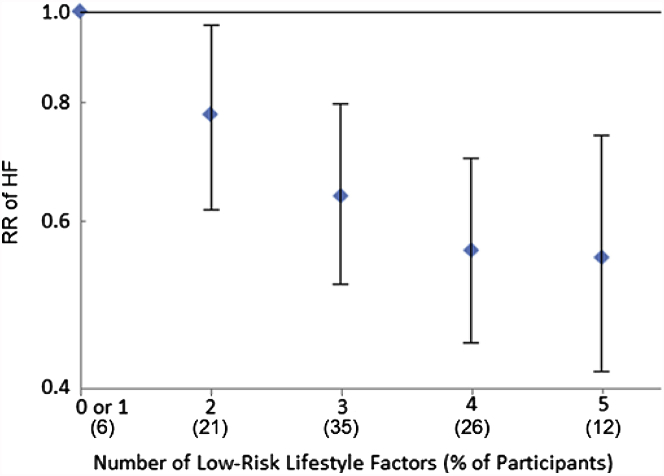
Risk of Incident HF According to Number of Low-Risk Lifestyle Factors in Older Adults (n = 4,490) Low-risk lifestyle factors included walking pace ≥2 mph, leisure activity ≥850 kcal/week, no current smoking, ≥1 drink/week of alcohol, and a body mass index <30 kg/m^2^. Risk estimates were adjusted for age (years), sex (male vs. female), race (Caucasian vs. non-Caucasian), enrollment site (4 clinics), education (less than high school, high school, more than high school), annual income (<$25,000, $25,000 to $49,999, >$50,000). HF = heart failure; RR = risk ratio.

After multivariable adjustment, smoking, modest alcohol intake, BMI, and waist circumference were each independently associated with incident HF, with 37%, 30%, 37%, and 20% lower risk among older adults in the lowest risk groups, respectively (Online Table 3). Because BMI was more strongly associated with HF than waist circumference, BMI (low risk group <30 kg/m^2^) was evaluated with other low-risk lifestyle factors (≥2 mph walking pace, leisure activity ≥845 kcal/week, no current smoking, ≥1 alcohol drink/week) to assess combined associations with incident HF. Compared with individuals with 0 or 1 low-risk lifestyle factors, participants had lower risk of HF if they had 2 (HR: 0.78; 95% CI: 0.62 to 0.97), 3 (HR: 0.64; 95% CI: 0.52 to 0.80), 4 (HR: 0.56; 0.44 to 0.70), or 5 (HR: 0.55; 95% CI: 0.42 to 0.74) low-risk lifestyle factors (Figure 1).

Compared with the low-risk group for each, each lifestyle factor was estimated to explain between 5% (smoking) and 18% (alcohol use) of the population risk of developing HF in older adults (Table 3). Lack of adherence to an overall healthy diet pattern had no associated attributable risk in older adults.

**Table 3:** Relative Risk of Incident HF by Lifestyle Factors and Adiposity in U.S. Older Adults (n = 4,490)

	% of Total Participants	Person-Years of Follow-Up	HF Cases	Multivariate Model HR (95% CI)∗	Multivariate + Lifestyle Model HR (95% CI)†	Population Attributable Risk HR (95% CI)‡
Healthy diet pattern§						
Lower 2 quintiles	36.1	19,605	583	1.00 (ref)	1.00 (ref)	
Upper 3 quintiles	63.9	32,244	797	0.91 (0.81 to 1.02)	0.98 (0.87 to 1.09)	0 (–3 to – 5)
Walking pace, mph						
<2	28.6	12,770	454	1.00 (ref)	1.00 (ref)	
≥2	71.4	39,079	926	0.72 (0.64 to 0.81)	0.80 (0.71 to 0.90)	7 (3 to 11)
Leisure activity, kcal/week‖						
<845	41.2	19,775	624	1.00 (ref)	1.0 (ref)	
≥845	58.8	32,074	756	0.72 (0.64 to 0.80)	0.78 (0.69 to 0.87)	11 (6 to 15)
Smoking						
Current	11.6	5,452	149	1.00 (ref)	1.00 (ref)	
Never or former	88.4	4,6397	1,231	0.77 (0.65 to 0.92)	0.71 (0.59 to 0.88)	5 (2 to 7)
Alcohol intake#, drink/week						
<1	72.3	36,803	1,040	1.00 (ref)	1.00 (ref)	
≥1	27.7	15,045	340	0.78 (0.68 to 0.88)	0.77 (0.67 to 0.88)	18 (9 to 26)
Body mass index, kg/m^2^						
≥30.0	19.2	10,050	336	1.00 (ref)	1.00 (ref)	
<30.0	80.8	41,799	1,044	0.66 (0.62 to 0.82)	0.70 (0.61 to 0.80)	8 (5 to 11)
Low-risk factors						
<4 low-risk factors	62.0	32,184	773	1.00 (ref)	1.00 (ref)	23 (14 to 26)
≥4	38.0	19,665	607	0.54 (0.40 to 0.66)	0.55 (0.42 to 0.74)	

Values are HR (95% CI) based on cumulatively updated exposures.

Abbreviations as in Table 2.

∗ The multivariate model was adjusted for age (years), sex (male vs. female), race (Caucasian vs. non-Caucasian), enrollment site (4 clinics), education (less than high school, high school, more than high school), annual income (<$25,000, $25,000 to $49,999, >$50,000).

† The multivariate + lifestyle model was mutually adjusted for other lifestyle factors in the table (categorization: healthy diet pattern [quintiles], leisure activity, kilocalories per week [quintiles], walking pace [<2, 2 to 3, >3 mph], smoking [never, former, current], alcohol intake [0, <1, 1 to 3, ≥3 drinks/week], body mass index [kilogram divided by square meter]).

‡ The population attributable risk is the percentage of new cases of heart failure in the population attributable to nonadherence to the low-risk lifestyle factor. Risk estimates from the multivariate + lifestyle model were used in calculating population attributable risk.

§ The Biologic pattern was used for the healthy diet score. The components of dietary pattern included fruits, vegetables, whole grains, fish/seafood, polyunsaturated to saturated fat ratio, nuts/seeds, red and processed meats, sugar-sweetened beverages, transfat, and sodium. Results were similar using DASH, AHEI, or AHA 2020 instead of the Biologic pattern.

‖ Kilocalorie cutoff approximates amount of energy expended by adhering to Centers for Disease Control and Prevention physical activity recommendations for older adults to achieve important health benefits.

# Alcohol intake was modest; <10% of adults consumed >2 drinks/week.

**Figure 2:**
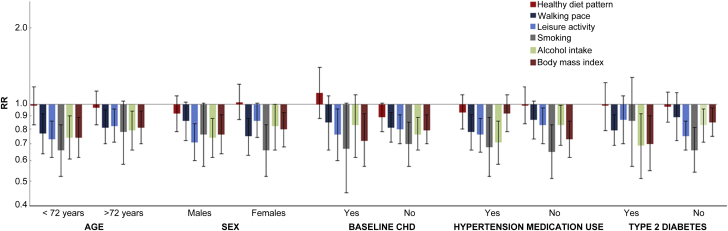
Risk of Incident HF for Low-Risk Lifestyle Factors in Major Subgroups of Older Adults (n = 4,490) Low-risk lifestyle factors included healthy diet pattern (upper 3 quintiles), walking pace ≥2 mph, leisure activity ≥850 kcal/week, no current smoking, ≥1 drink/week of alcohol intake, and a body mass index <30 kg/m^2^. Multivariate models for each lifestyle factor were adjusted for age (years), sex (male vs. female), race (Caucasian vs. non-Caucasian), enrollment site (4 clinics), education (less than high school, high school, more than high school), annual income (<$25,000, $25,000 to $49,999, >$50,000) and mutually adjusted for other lifestyle factors in the figure. None of the differences were significant after correcting for multiple comparisons. CHD = coronary heart disease; other abbreviations as in Figure 1.

Heterogeneity in the association between diet and HF was not observed by any of the potential effect modifiers (p_interaction_ >0.05) (Online Table 4), although the AHA 2020 diet pattern was associated with a trend toward lower HF risk in African Americans (p_interaction_ = 0.07) and those without baseline CHD (p_interaction_ = 0.06; HR: 0.86; 95% CI 0.70 to 1.06). After Bonferroni correction, there was also little evidence for heterogeneity by age, sex, baseline CHD, treated hypertension, and diabetes for the association of nondietary lifestyle factors with HF (Figure 2).

In the secondary analyses that evaluated individual dietary components, including fruits, vegetables, whole grains, fish, polyunsaturated to saturated fat ratio, nuts, red meat, processed meat, sugar-sweetened beverages, and transfat, no component was significantly associated with incident HF (Online Table 5). After adjusting for demographic and lifestyle variables, sodium was associated with a 19% increased risk of incident HF in the highest versus lowest quintile of intake. When potential mediators were added to the model, the association was slightly attenuated (highest quintile HR: 1.15; 95% CI 0.96 to 1.36; p_trend_ = 0.05), with diagnosed diabetes having the largest influence on mitigating the sodium–HF association.

All results were similar if we excluded participants with prevalent CHD, except for a stronger positive association between dietary sodium and incident CHD (p_trend_ = 0.014) (Online Tables 6 to 8). In additional sensitivity analysis restricted to adults with self-perceived excellent, very good, or good health, associations were not materially altered (Online Tables 9 and 10). In contrast, inverse associations of walking pace and leisure activity were attenuated in self-perceived healthier participants (Online Table 10). Adjustment for baseline N-terminal pro–B-type natriuretic peptide had minimal influence on associations for diet patterns, walking pace, smoking, and alcohol intake (Online Table 11).

## Discussion

In this large, prospective cohort of older U.S. adults, lifestyle factors, including moderate alcohol use, physical activity, not smoking, and avoiding obesity late in life, were each independently associated with a lower risk of incident HF. Participants with ≥4 of these healthy lifestyle factors, compared with none, had a 45% lower risk of developing HF. Among different physical activity measures, walking pace and leisure activity, but not exercise intensity, were each independently associated with lower risk. If associations were causal, our results suggested that moderate physical activity (>845 kcal/week in leisure activity) and higher walking speed (>3 mph), rather than high-intensity exercise, might be most useful in older adults for HF prevention. A second important, and unexpected, finding, was that overall dietary patterns were not associated with HF. However, higher sodium intake was associated with trends toward increased risk in the overall cohort, and significantly increased risk among those without CHD at baseline. Our work provided an assessment of the relative importance and burden of major lifestyle factors in the development of HF in older adults, the fastest growing segment of the U.S. population.

The association of physical activity with lower HF is mechanistically plausible because of the benefits on endothelial function, autonomic function, nitric oxide bioavailability, and progenitor cell mobilization 17, 18. Benefits may be mediated through prevention of hypertension, left ventricular hypertrophy, obesity, CHD, and type 2 diabetes, which are all major risk factors for HF (17). Our findings that moderate energy expenditure through leisure activity is associated with a lower HF risk, as well as the lack of independent association for exercise intensity, are consistent with recent prospective analyses that showed little or no additional benefit accrued from vigorous over moderate activity for prevention of hypertension, CHD, and diabetes 19, 20. Physical activity was no longer associated with HF in sensitivity analyses restricted to individuals with better self-perceived health. This attenuation could reflect better adjustment due to confounding from subclinical morbidity. Alternatively, the attenuation could reflect over-adjustment (adjustment for a major mediator of the effect), because physical activity increases self-perceived health in older adults, including benefits on mental well-being, physical well-being, quality of life (21), and reduced hospitalizations among patients with HF (22).

Moderate alcohol use is associated with lower HF risk in most longitudinal analyses (23). Although the upper limit of the low-risk alcohol intake category in our analysis was unconstrained, alcohol use in the CHS was low, with only 25% of participants consuming >1 drink/week, and <10% consuming >2 drinks/week. Heavy alcohol use induces alcoholic cardiomyopathy (24) and increases risk of HF, whereas modest use improves endothelial function and increases plasma atrial natriuretic peptide (25). In the absence of large randomized trials of moderate alcohol use and incident HF, in addition to risk of abuse and other adverse health consequences, it would be premature to recommend alcohol intake for public health prevention of HF. However, our findings support modest use among current alcohol users without contraindications.

Avoidance of smoking and obesity were also independently associated with lower HF risk. The observed magnitude of association for smoking was similar to that observed in the Coronary Artery Surgery Study, in which smoking was associated with a 47% increased risk of HF (26). Associations of obesity with incident HF may be mediated through hypertension, CHD, type 2 diabetes, and sleep apnea (27); key postulated mechanisms include increases in atherogenic lipids, cardiac preload and afterload, and neurohormonal disruption (17).

Although robust evidence from longitudinal studies and randomized trials showed that healthful dietary patterns reduced major HF risk factors, such as high blood pressure and CHD 13, 14, 28, 29, no known large trials and few observational studies directly linked dietary patterns and their components with incident HF; these studies had mixed results. Our findings of no association of overall dietary patterns with HF differed from 2 Swedish cohorts, in which adherence to DASH was associated with a 22% to 37% lower risk of HF in the highest quartiles 8, 9. The absolute incidence of HF in these cohorts was approximately 8-fold lower than in the CHS, and these previous studies only captured HF events that resulted in hospitalization or death. In the CHS, incident HF was adjudicated by a centralized CHS committee that used all available outpatient and inpatient data, which more completely captured HF, including those treated on an outpatient basis. The CHS also included only elderly participants, compared with middle-aged participants in the Swedish cohorts. These differences could account for the varied findings; our results highlighted the need for further study of dietary factors, including sodium, and incident HF.

Our analysis had several strengths. Demographic characteristics, lifestyle factors, and HF events were prospectively recorded with little loss to follow-up over 21.5 years. Repeated assessments of lifestyle factors allowed for cumulative updating, minimizing misclassification. Multiple dietary patterns were investigated, including 2 established patterns associated with reduced hypertension and CHD (DASH and AHEI) and 2 additional biologically derived patterns (Biologic and AHA 2020). The lack of association observed across all patterns for incident HF implied that our dietary findings were robust. We restricted our sensitivity analyses to those without prevalent CHD, which reduced confounding by indication and corroborated our main findings. We used time-varying covariates to adjust for time-dependent confounding. A large number of validated HF cases provided sufficient power to detect associations, including across multiple risk categories. Participants were selected randomly and enrolled from Medicare eligibility lists in several U.S. communities, which provided a population-based sample of older adults and increased generalizability.

### Study limitations

Potential limitations should be considered. Although we adjusted for major demographics and lifestyle factors, residual confounding by unknown or unmeasured factors might be present. Associations of dietary patterns and physical activity might be mediated through adiposity, hypertension, and prevalent CHD; therefore, analyses adjusted for these factors might underestimate the impact of lifestyle on HF. However, we performed stratified analyses and tests for interactions for diet and lifestyle factors by these potential mediating factors. Some misclassification of lifestyle factors was inevitable, especially in those that were assessed via self-report, as well as in those analyzed in categories or dichotomously, which would likely attenuate findings toward the null and underestimate the true magnitude of associations. We also highlight the need to better understand and integrate the determinants and relative contribution of lifestyle and other risk factors for HF beyond the scope of this work, including congenital defects, cardiomyopathies, drugs and/or toxins, renal dysfunction, and genetic risk predictors.

## Conclusions

Our findings suggested that adherence to a few modifiable risk factors, including physical activity, moderate alcohol use, not smoking, and avoiding obesity, halved the risk of incident HF later in life. Although overall dietary patterns were not associated with lower HF risk in this cohort, adherence to a healthy diet remains crucial for prevention of other cardiometabolic diseases, including hypertension, type 2 diabetes, and CHD. Our results also underscored the importance of further investigating specific dietary determinants, such as sodium, and physical activity type, duration, and frequency in future studies and trials for HF prevention among older adults.

Perspectives **COMPETENCY IN MEDICAL KNOWLEDGE:** In older adults, the risk of developing HF can be cut in half by adherence to key protective lifestyle factors, including modest physical activity, in addition to not smoking and maintaining a healthy weight.

**TRANSLATIONAL OUTLOOK:** Randomized trials investigating physical activity type, duration, and frequency in more detail for the prevention of HF in older adults are needed.
