# Supplementary material for: Contribution of Major Lifestyle Risk Factors for Incident Heart Failure in Older Adults: The Cardiovascular Health Study
Source: JACC Heart Fail. 2015 Jul;3(7):520–8. doi: 10.1016/j.jchf.2015.02.009 (PMC4508377; doi:10.1016/j.jchf.2015.02.009)
Supplement: Online Tables 1–11 [file mmc1.docx]

**SUPPLEMENTARY TABLE 1**. Dietary pattern scoring algorithms^a^

| **DIETARY PATTERN** | **RANGE OF INTAKE** | **SCORE** |
| --- | --- | --- |
| **DASH^b^**  (1) Low-fat dairy  (2) Fruits  (3) Vegetables  (4) Nuts and legumes  (5) Whole grains  (6) Red & processed meats  (7) SSB  (8) Sodium | Q1-5  Q1-5  Q1-5  Q1-5  Q1-5  Q1-5  Q1-5  Q1-5 | 1-5  1-5  1-5  1-5  1-5  5-1  5-1  5-1 |
| **AHEI^c^**  (1) Fruit (servings/day)  (2) Vegetables (servings/day)  (3) Nuts and soy protein (servings/day)  (4) Cereal fiber (g/day)  (5) PUFA:SF ratio  (6) Trans fat (% energy)  (7) Alcohol (drinks/day)  (8) Long-term multivitamin use (years)  (9) White meat: red meat ratio | 0 to ≥ 4  0 to ≥ 5  0 to ≥ 1  0 to ≥ 15  ≤ 0.1 to ≥1  ≤ 0.5 to ≥ 4  0 or > 2.5 vs. 0.5 to 1.5 (women)  0 or > 3.5 vs. 1.5 to 2.5 (men)  0 to ≥ 5  0 to ≥ 4 | 0-10  0-10  0-10  0-10  0-10  10-0  10 for optimal range  10 for optimal range  2.5-7.5  0-10 |
| **BIOLOGIC^d^**  (1) Fruits  (2) Vegetables  (3) Whole grains  (4) Fish/seafood  (5) Polyunsaturated: saturated fat ratio (PUFA: SF ratio)  (6) Nuts/seeds  (7) Red & processed meats  (8) Sugar-sweetened beverages (SSB)  (9) Trans fat  (10) Sodium | Q1-5  Q1-5  Q1-5  Q1-5  Q1-5  Q1-5  Q1-5  Q1-5  Q1-5  Q1-5 | 1-5  1-5  1-5  1-5  1-5  1-5  5-1  5-1  5-1  5-1 |
| **AHA dietary 2020 goals^e^**  (1) Fruit & vegetables (cups/day)^3^  (2) Fish (3.5oz servings/week)  (3) Fiber-rich whole grains (1oz servings/day)  (4) Nuts, legumes, and seeds (servings/week)  (5) Sodium (mg/day)  (6) SSB (oz/week)  (7) Processed meats (servings/week)  (8) Saturated fat (% total energy intake) | 0 to ≥ 4.5  0 to ≥ 2  0 to ≥ 3  0 to ≥ 4  ≤ 1500 or >4500  ≤ 36 or >84  ≤ 2 or >7  ≤ 7 or >20 | 0-10  0-10  0-10  0-10  10-0  10-0  10-0  10-0 |

^a^ Intakes were energy-adjusted using the residual method ^b^ The DASH score, developed by Fung and Chiuve, is comprised of five dietary components associated with a lower risk of hypertension (fruits, vegetables, nuts & legumes, low-fat dairy, and whole grains) and three associated with increased risk (red & processed meats, sugar-sweetened beverages, sodium) (15). ^c^ The aHEI score was developed by McCullough et al. (16) by revising the HEI to combine fruits and vegetables, eliminate total grains, and add nuts and cereal fiber. Optimal intakes were assigned a score of 10 and least optimal intakes a score of 0; intermediate intakes were scored in a linear proportionate fashion between 1 and 9 ^d^ The Biologic pattern was constructed based on a priori knowledge of specific dietary components associated with risk of CHD, hypertension, and/or diabetes, which are major risk factors for HF; these include fruits, vegetables, whole grains, fish/seafood, polyunsaturated fatty acids (excluding seafood omega-3 fatty acids), nuts/seeds, red and processed meats, sugar-sweetened beverages, trans-fatty acids, and sodium. Potatoes were excluded from vegetable intake. For each dietary component, we created energy-adjusted quintiles and assigned each individual a score of 1 to 5 according to the quintile of intake, with 5 representing the most favorable quintile. ^e^ The AHA 2020 score was constructed based on recommended dietary components from AHA’s strategic impact goals for improving cardiovascular health by 2020, including fruits & vegetables, fish, fiber-rich whole grains, nuts, legumes & seeds, sodium, sugar-sweetened beverages, processed meats, and saturated fat. Potatoes were excluded from vegetable intake. For each dietary component, we created scores based on whether an individual met AHA recommended intakes; as intake goals were expressed for a 2000 kcal/day diet, we followed the AHA suggestion to scale dietary factors accordingly for other levels of caloric intake (13). Optimal intakes were assigned a score of 10 and least optimal intakes a score of 0; intermediate intakes were scored in a linear proportionate fashion between 1 and 9.

**SUPPLEMENTARY TABLE 2**. Independence of physical activity measures on the development of HF in older US adults (n=4490)

|  | Multivariate^a^ | Multivariate + lifestyle^b^ | Multivariate + mediator^c^ |
| --- | --- | --- | --- |
| Exercise intensity  None  Low  Moderate  High | 1.0 (ref)  0.77 (0.63-0.93)  0.64 (0.52-0.79)  0.68 (0.53-0.88) | 1.0 (ref)  0.94 (0.76-1.17)  0.86 (0.68-1.09)  0.98 (0.74-1.30) | 1.0 (ref)  0.97 (0.78-1.20)  0.91 (0.72-1.15)  1.05 (0.79-1.40) |
| Walking pace  < 2 mph  2-3 mph  > 3 mph | 1.0 (ref)  0.77 (0.68-0.87)  0.63 (0.55-0.73) | 1.0 (ref)  0.85 (0.74-0.96)  0.75 (0.64-0.88) | 1.0 (ref)  0.87 (0.76-0.99)  0.78 (0.67-0.91) |
|  |  |  |  |
| Leisure activity, kcal/wk |  |  |  |
| Q1 (0-330)  Q2 (331-844)  Q3 (845-1530)  Q4 (1531-2945)  Q5 (≥ 2946) | 1.0 (ref)  0.90 (0.78-1.08)  0.68 (0.58-0.81)  0.71 (0.60-0.84)  0.67 (0.57-0.80) | 1.0 (ref)  0.97 (0.82-1.15)  0.72 (0.60-0.87)  0.77 (0.64-0.93)  0.75 (0.61-0.92) | 1.0 (ref)  1.01 (0.85-1.21)  0.76 (0.63-0.92)  0.79 (0.65-0.96)  0.80 (0.65-0.98) |
| Walking distance (blocks/wk)  0-6  7-12  13-30  31-70  >70 | 1.0 (ref)  0.75 (0.64-0.89)  0.73 (0.61-0.86)  0.73 (0.61-0.86)  0.78 (0.66-0.93) | 1.0 (ref)  0.82 (0.69-0.97)  0.84 (0.71-1.00)  0.87 (0.73-1.04)  1.02 (0.85-1.22) | 1.0 (ref)  0.88 (0.74-1.04)  0.88 (0.74-1.05)  0.90 (0.75-1.08)  1.05 (0.87-1.26) |

^a^ Multivariate model was adjusted for age (years), sex (male vs. female), race (Caucasian vs. non-Caucasian), enrollment site (4 clinics), education (< high school, =high school, >high school), annual income (<$25 000, $25 000-$49 999, >$50 000)  ^b^ Multivariate + lifestyle model was mutually adjusted for other lifestyle factors [categorization: exercise intensity (none, low, moderate, high), walking pace (<2 mph, 2-3mph, >3mph), leisure activity, kcal/week (quintiles), walking distance, blocks/week (quintiles), healthy diet score (quintiles), smoking (never, former, current), alcohol intake (none, <1 drink/wk, 1-3 drink/wk, ≥3 drink/wk), body mass index (kg/m^2^)]  ^c^ Multivariate + mediator model was adjusted for all covariates in the multivariate + lifestyle model, with additional adjustment for potential mediators including body mass index (kg/m^2^), prevalent treated hypertension (yes vs. no), prevalent diabetes mellitus (yes vs. no), prevalent coronary heart disease (yes vs. no)

**SUPPLEMENTARY TABLE 3.** Relative risk of incident HF by lifestyle risk factors and adiposity in older US adults (n=4490)^a^

| HR (95% CI) .  % of total Person-years Cases Multivariate Multivariate + lifestyle Multivariate +mediator  participants of follow-up Model^b^ Model^c^ Model^d^ | | | | | | |
| --- | --- | --- | --- | --- | --- | --- |
| Healthy diet pattern^a^  Q1  Q2  Q3  Q4  Q5 | 19.7  20.7  21.4  20.7  17.4 | 9702  9903  10539  10632  11073 | 277  306  276  278  243 | 1.0 (ref)  1.14 (0.97-1.34)  1.00 (0.84-1.19)  1.01 (0.85-1.20)  0.91 (0.75-.1.09) | 1.0 (ref)  1.22 (1.03-1.44)  1.08 (0.91-1.28)  1.12 (0.94-1.34)  1.05 (0.87-1.27) | 1.0 (ref)  1.19 (1.00-1.40)  1.05 (0.88-1.25)  1.08 (0.91-1.29)  0.99 (0.82-1.19) |
| Walking pace  < 2 mph  2-3 mph  > 3 mph | 28.6  43.1  28.3 | 12770  22215  16865 | 454  585  341 | 1.0 (ref)  0.77 (0.68-0.87)  0.63 (0.55-0.73) | 1.0 (ref)  0.83 (0.73-0.94)  0.74 (0.63-0.86) | 1.0 (ref)  0.87 (0.76-0.99)  0.79 (0.68-0.92) |
|  |  |  |  |  |  |  |
| Leisure activity, kcal/wk |  |  |  |  |  |  |
| Q1 (0-330)  Q2 (331-844)  Q3 (845-1530)  Q4 (1531-2945)  Q5 (≥ 2946) | 20.9  20.3  19.7  19.7  19.3 | 9638  10137  10523  10616  10933 | 325  299  250  255  251 | 1.0 (ref)  0.92 (0.78-1.08)  0.68 (0.58-0.81)  0.71 (0.60-0.84)  0.67 (0.57-0.80) | 1.0 (ref)  0.96 (0.82-1.13)  0.73 (0.62-0.87)  0.79 (0.66-0.93)  0.76 (0.64-0.91) | 1.0 (ref)  1.00 (0.85-1.18)  0.78 (0.65-0.92)  0.81 (0.68-0.96)  0.82 (0.69-0.98) |
| Smoking  Never  Former  Current  Alcohol^f^  None  <1 drink/wk  1-3 drink/wk  ≥ 3 drink/wk | 46.2  42.2  11.6  48.1  24.2  10.6  17.1 | 25213  21183  5452  23672  13131  5962  9084 | 614  617  149  726  314  136  204 | 0.70 (0.59-0.84)  0.85 (0.72-1.03)  1.0 (ref)  1.0 (ref)  0.80 (0.70-0.92)  0.71 (0.58-0.85)  0.71 (0.61-0.84) | 0.63 (0.53-0.77)  0.80 (0.66-0.96)  1.0 (ref)  1.0 (ref)  0.78 (0.69-0.90)  0.68 (0.56-0.83)  0.70 (0.60-0.84) | 0.60 (0.49-0.72)  0.72 (0.60-0.87)  1.0 (ref)  1.0 (ref)  0.82 (0.71-0.94)  0.74 (0.61-0.89)  0.74 (0.63-0.88) |
|  |  |  |  |  |  |  |
| Body mass index  < 22.0  22.0-24.9  25.0-29.9  ≥ 30.0 | 13.9  24.1  42.7  19.2 | 7077  12611  22110  10050 | 160  289  595  336 | 0.59 (0.49-0.72)  0.61 (0.52-0.72)  0.72 (0.63-0.82)  1.0 (ref) | 0.63 (0.52-0.76)  0.65 (0.55-0.77)  0.75 (0.65-0.86)  1.0 (ref) | 0.73 (0.59-0.88)  0.71 (0.60-0.84)  0.80 (0.69-0.92)  1.0 (ref) |
|  |  |  |  |  |  |  |
| Waist circumference^g^ < 88cm women, < 92cm men  ≥ 88cm women, ≥ 92cm men | 35.9  64.1 | 19379  32470 | 427  953 | 0.76 (0.68-0.86)  1.0 (ref) | 0.80 (0.71-0.90)  1.0 (ref) | 0.86 (0.77-0.97)  1.0 (ref) |

^a^ Data are HR (95% CI) based on cumulatively updated exposures ^b^ Multivariate model was adjusted for age (years), sex (male vs. female), race (Caucasian vs. non-Caucasian), enrollment site (4 clinics), education (< high school, =high school, >high school), annual income (<$25 000, $25 000-$49 999, >$50 000) ^c^ Multivariate + lifestyle model was mutually adjusted for other lifestyle factors in the table [categorization: healthy diet pattern (quintiles), leisure activity, kcal/wk (quintiles), walking pace (<2 mph, 2-3mph, >3mph), smoking (never, former, current), alcohol intake (none, <1 drink/wk, 1-3 drink/wk, ≥3 drink/wk), body mass index (<22, 22-24.9, 25-29.9, ≥30kg/m^2^]  ^d^ Multivariate + mediator model was adjusted for all covariates in the multivariate + lifestyle model, with additional adjustment for potential mediators including body mass index (kg/m^2^), prevalent treated hypertension (yes vs. no), prevalent diabetes mellitus (yes vs. no), prevalent coronary heart disease (yes vs. no) ^e^ The Biologic pattern was used for the healthy diet score. The components of dietary pattern included fruits, vegetables, whole grains, fish/seafood, polyunsaturated to saturated fat ratio, nuts/seeds, red and processed meats, sugar-sweetened beverages, trans fat, and sodium. Results were similar using DASH, AHEI, or AHA 2020 instead of the Biologic pattern ^f^ Alcohol intake was modest; <10% of adults consumed > 2 drinks/week ^g^ The low-risk classifications were based on the observed distribution of risk factors within the CHS cohort and current recommendations, when available; for instance, low risk for waist circumference was defined as less than 88 cm in women or 92 cm in men based on Adult Treatment Panel III criteria

**SUPPLEMENTARY TABLE 4.** Effect modification of relative risk of incident HF by diet-quality scores in older adults (n=4490)^a^

| Potential effect modifier | BIOLOGIC  HR (95% CI) | *p* ^b^ | DASH  HR (95% CI) | *p* | AHEI  HR (95% CI) | *p* | AHA 2020  HR (95% CI) | *p* |
| --- | --- | --- | --- | --- | --- | --- | --- | --- |
| Age  < 72y  ≥ 72y | 1.03 (0.78-1.36)  0.85 (0.69-1.11) | 0.36 | 1.00 (0.76-1.32)  0.96 (0.74-1.25) | 0.82 | 0.81 (0.61-1.08)  0.89 (0.68-1.18) | 0.34 | 0.79 (0.60-1.05)  1.02 (0.78-1.32) | 0.21 |
| Sex  Male  Female | 0.98 (0.75-1.29)  0.95 (0.71-1.27) | 0.62 | 0.93 (0.70-1.24)  1.17 (0.91-1.51) | 0.44 | 0.77 (0.57-1.03)  0.99 (0.76-1.29) | 0.20 | 0.77 (0.57-1.03)  0.94 (0.72-1.22) | 0.32 |
| Race  Caucasian  African-American | 0.99 (0.84-1.25)  0.98 (0.52-1.84) | 0.80 | 1.08 (0.89-1.31)  0.89 (0.50-1.60) | 0.42 | 0.89 (0.73-1.09)  0.98 (0.55-1.73) | 0.32 | 1.00 (0.84-1.19)  0.57 (0.28-1.13) | 0.07 |
| BMI  < 25 kg/m^2^  ≥ 25 kg/m^2^ | 0.75 (0.54-1.01)  1.15 (0.91-1.45) | 0.18 | 1.07 (0.79-1.46)  1.03 (0.82-1.29) | 0.70 | 0.68 (0.50-0.93)  1.03 (0.81-1.31) | 0.10 | 0.93 (0.69-1.26)  0.95 (0.76-1.20) | 0.64 |
| Treated hypertension  Yes  No | 1.03 (0.80-1.33)  0.93 (0.70-1.22) | 0.65 | 1.06 (0.82-1.36)  1.04 (0.80-1.35) | 0.61 | 0.89 (0.68-1.16)  0.90 (0.68-1.19) | 0.89 | 0.86 (0.67-1.11)  1.07 (0.83-1.39) | 0.22 |
| Diabetes  Yes  No | 0.91 (0.64-1.30)  1.04 (0.83-1.29) | 0.62 | 0.96 (0.66-1.38)  1.09 (0.88-1.35) | 0.50 | 0.84 (0.57-1.24)  0.94 (0.76-1.18) | 0.40 | 0.90 (0.63-1.28)  0.99 (0.80-1.22) | 0.37 |
| CHD  Yes  No | 1.28 (0.89-1.85)  0.89 (0.71-1.10) | 0.09 | 1.13 (0.77-1.65)  1.03 (0.84-1.27) | 0.56 | 0.97 (0.66-1.43)  0.86 (0.69-1.08) | 0.48 | 1.28 (0.89-1.83)  0.86 (0.70-1.06) | 0.06 |

^a^ Data are extreme-quintile (Q5 vs. Q1) HR (95% CI) based on cumulatively averaged Biologic score, Dietary Approaches to Stop Hypertension (DASH) score, the Alternative Healthy Eating Index (AHEI) score, and the American Heart Association dietary 2020 goals (AHA 2020) score ^b^ *p*(interaction) was assessed using Wald tests. Interaction terms were added individually to a model adjusted for age (years), sex (male vs. female), race (Caucasian vs. non-Caucasian), enrollment site (4 clinics), education (< high school, =high school, >high school), annual income (<$25 000, $25 000-$49 999, >$50 000), total kcal expended (quintiles), walking pace (<2 mph, 2-3mph, >3mph), smoking (never, former, current), alcohol intake (none, <1 drink/wk, 1-2 drink/wk, ≥3 drink/wk), body mass index (kg/m2), prevalent treated hypertension (yes vs. no), prevalent diabetes mellitus (yes vs. no), prevalent coronary heart disease (yes vs. no)

**SUPPLEMENTARY TABLE 5.** Relative risk of incident HF by quintiles of dietary components in older adults (n=4490)^a^

| . Quintiles of intake .  1 2 3 4 5 *P*(trend)^b^ | | | | | | |
| --- | --- | --- | --- | --- | --- | --- |
| Fruits  Multivariate^c^  + Mediator adjusted^d^ | 1.0 (ref)  1.0 (ref) | 0.81 (0.68-0.96)  0.80 (0.67-0.95) | 1.04 (0.88-1.22)  1.02 (0.86-1.20) | 1.12 (0.95-1.31)  1.07 (0.90-1.26) | 0.96 (0.81-1.14)  0.92 (0.77-1.09) | 0.21  0.56 |
|  |  |  |  |  |  |  |
| Vegetables  Multivariate  + Mediator adjusted | 1.0 (ref)  1.0 (ref) | 1.03 (0.87-1.21)  1.00 (0.85-1.18) | 1.08 (0.92-1.28)  1.06 (0.89-1.25) | 1.09 (0.91-1.29)  1.05 (0.89-1.25) | 1.21 (1.01-1.43)  1.18 (0.99-1.41) | 0.10  0.16 |
|  |  |  |  |  |  |  |
| Whole grains |  |  |  |  |  |  |
| Multivariate  + Mediator adjusted | 1.0 (ref)  1.0 (ref) | 0.90 (0.76-1.07)  0.89 (0.75-1.05) | 1.12 (0.95-1.32)  1.14 (0.97-1.34) | 1.05 (0.89-1.25)  1.04 (0.88-1.23) | 1.00 (0.84-1.18)  1.00 (0.85-1.19) | 0.48  0.38 |
|  |  |  |  |  |  |  |
| Fish |  |  |  |  |  |  |
| Multivariate  + Mediator adjusted | 1.0 (ref)  1.0 (ref) | 1.11 (0.94-1.31)  1.07 (0.90-1.26) | 1.12 (0.95-1.32)  1.11 (0.94-1.31) | 1.10 (0.93-1.31)  1.06 (0.89-1.26) | 1.00 (0.85-1.19)  0.98 (0.82-1.18) | 0.83  0.57 |
|  |  |  |  |  |  |  |
| PUFA/SF ratio^e^ |  |  |  |  |  |  |
| Multivariate  + Mediator adjusted | 1.0 (ref)  1.0 (ref) | 1.06 (0.90-1.25)  1.05 (0.89-1.24) | 1.08 (0.92-1.28)  1.08 (0.92-1.28) | 0.93 (0.79-1.10)  0.94 (0.89-1.12) | 0.96 (0.81-1.14)  0.98 (0.82-1.16) | 0.36  0.51 |
|  |  |  |  |  |  |  |
| Nuts/seeds  Multivariate  + Mediator adjusted | 1.0 (ref)  1.0 (ref) | 0.91 (0.77-1.07)  0.87 (0.74-1.03) | 0.89 (0.75-1.05)  0.87 (0.73-1.03) | 0.91 (0.77-1.08)  0.91 (0.77-1.07) | 0.89 (0.76-1.05)  0.88 (0.74-1.04) | 0.66  0.65 |
|  |  |  |  |  |  |  |
| Red & processed meat  Multivariate  + Mediator adjusted | 1.0 (ref)  1.0 (ref) | 1.11 (0.94-1.32)  1.10 (0.93-1.31) | 1.03 (0.87-1.23)  1.03 (0.86-1.22) | 1.09 (0.92-1.29)  1.10 (0.93-1.30) | 1.15 (0.96-1.36)  1.09 (0.91-1.29) | 0.19  0.46 |
| Red meat (unprocessed)^f^  Multivariate  + Mediator adjusted | 1.0 (ref)  1.0 (ref) | 0.78 (0.66-0.93)  0.77 (0.65-0.91) | 0.92 (0.78-1.09)  0.92 (0.78-1.09) | 0.91 (0.77-1.07)  0.91 (0.77-1.07) | 0.93 (0.79-1.10)  0.94 (0.80-1.10) | 0.47  0.44 |
| Processed meat^g^  Multivariate  + Mediator adjusted  Sugar-sweetened beverages  Multivariate  + Mediator adjusted | 1.0 (ref)  1.0 (ref)  1.0 (ref)  1.0 (ref) | 1.07 (0.92-1.23)  1.01 (0.95-1.27)  0.93 (0.78-1.10)  0.92 (0.77-1.09) | 1.10 (0.83-1.45)  1.11 (1.84-1.47)  0.91 (0.77-1.09)  0.94 (0.79-1.12) | 1.10 (0.83-1.45)  1.12 (0.85-1.48)  0.95 (0.80-1.13)  0.96 (0.81-1.14) | 1.15 (1.87-1.52)  1.21 (0.92-1.60)  0.96 (0.81-1.14)  0.97 (0.81-1.15) | 0.30  0.19  0.28  0.32 |
| Trans fat  Multivariate  + Mediator adjusted | 1.0 (ref)  1.0 (ref) | 1.08 (0.91-1.29)  1.09 (0.92-1.32) | 1.16 (0.97-1.38)  1.16 (0.98-1.39) | 1.21 (1.02-1.43)  1.20 (1.01-1.43) | 1.01 (0.84-1.20)  1.04 (0.87-1.25) | 0.94  0.65 |
| Sodium  Multivariate  + Mediator adjusted | 1.0 (ref)  1.0 (ref) | 1.11 (0.93-1.32)  1.10 (0.92-1.30) | 1.18 (0.99-1.40)  1.14 (0.96-1.36) | 1.26 (1.06-1.49)  1.23 (1.04-1.46) | 1.19 (1.00-1.41)  1.15 (0.96-1.36) | **0.014**  **0.05** |

^a^ Data are HR (95% CI) based on cumulatively updated intake. The foods were components of the Biologic dietary pattern ^b^ Linear trend was tested by assigning the median value to participants in each quintile and entering this into the model as a continuous variable ^c^ Multivariate model: adjusted for age (years), sex (male vs. female), race (Caucasian vs. non-Caucasian), enrollment site (4 clinics), education (< high school, =high school, >high school), annual income (<$25 000, $25 000-$49 999, >$50 000), total kcal expended (quintiles), walking pace (<2 mph, 2-3mph, >3mph), smoking (never, former, current), alcohol intake (none, <1 drink/wk, 1-2 drink/wk, ≥3 drink/wk) ^d^ + Mediator adjusted: Multivariate model + additional adjustment for potential mediators, including body mass index (kg/m^2^), prevalent treated hypertension (yes vs. no), prevalent diabetes mellitus (yes vs. no), prevalent coronary heart disease (yes vs. no) ^e^ Polyunsaturated fat to saturated fat ratio ^f^ Red meat (unprocessed) was defined as beef steaks/roasts, beef stew, and pork chops/roasts ^g^ Processed meat was defined as hot dogs, ham, lunch meat, bacon, and sausage

**SUPPLEMENTARY TABLE 6.** Hazard ratios (95% CI) for incident HF by quintiles of diet-quality scores in older US adults without prevalent CHD (n=3734)^a^

| .Quintiles of diet-quality scores .  1 2 3 4 5 *P*(trend)^b^ | | | | | | |
| --- | --- | --- | --- | --- | --- | --- |
| Biologic^c^  Cases/person-years  Multivariate^g^  + Mediator adjusted^h^ | 209/8390  1.0 (ref)  1.0 (ref) | 253/8698  1.25 (1.04-1.51)  1.21 (1.00-1.45) | 209/9051  1.04 (0.86-1.27)  1.00 (0.83-1.23) | 210/9184  1.04 (0.85-1.27)  1.01 (0.83-1.23) | 175/9553  0.92 (0.74-1.15)  0.89 (0.71-1.10) | 0.18  0.10 |
| DASH^d^  Cases/person-years  Multivariate  + Mediator adjusted | 217/8954  1.0 (ref)  1.0 (ref) | 262/10398  1.16 (0.97-1.39)  1.14 (0.96-1.37) | 206/8764  1.12 (0.92-1.35)  1.08 (0.89-1.30) | 192/8486  1.11 (0.91-1.36)  1.07 (0.87-1.30) | 179/8273  1.08 (0.87-1.33)  1.03 (0.84-1.27) | 0.56  0.91 |
| AHEI^e^  Cases/person-years  Multivariate  + Mediator adjusted | 226/8199  1.0 (ref)  1.0 (ref) | 229/8455  1.06 (0.88-1.27)  1.00 (0.83-1.20) | 234/9591  1.00 (0.83-1.21)  0.97 (0.80-1.17) | 193/8850  0.95 (0.77-1.17)  0.93 (0.76-1.14) | 174/9780  0.89 (0.71-1.10)  0.86 (0.69-1.08) | 0.18  0.16 |
| AHA 2020^f^  Cases/person-years  Multivariate  + Mediator adjusted | 281/9766  1.0 (ref)  1.0 (ref) | 283/10569  1.06 (0.88-1.27)  0.98 (0.81-1.18) | 309/10368  1.00 (0.83-1.21)  1.18 (0.98-1.42) | 267/11100  0.95 (0.77-1.17)  0.98 (0.80-1.20) | 240/10618  0.89 (0.71-1.11)  0.86 (0.70-1.06) | 0.61  0.32 |

^a^ Data are HR (95% CI) based on cumulatively averaged Biologic score, Dietary Approaches to Stop Hypertension (DASH) score, the Alternative Healthy Eating Index (AHEI) score, and the American Heart Association dietary 2020 goals (AHA 2020) score ^b^ Linear trend was tested by assigning the median value to participants in each quintile and entering this into the model as a continuous variable ^c^ Biologic dietary pattern was comprised of ten components: 1.) fruits 2.) vegetables 3.) whole grains 4.) fish 5.) polyunsaturated to saturated fat ratio 6.) nuts/seeds 7.) red & processed meats 8.) sugar-sweetened beverages 9.) trans fat 10.) sodium. For scoring of dietary patterns, see **Supplementary Table 1**. ^d^ DASH dietary pattern was comprised of eight components: 1.) low-fat dairy 2.) fruits 3.) vegetables 4.) nuts & legumes 5.) whole grains 6.) red & processed meats 7.) sugar-sweetened beverages 8.) sodium ^e^ AHEI dietary pattern was comprised of nine components: 1.) fruits 2.) vegetables 3.) nuts & soy protein 4.) cereal fiber 5.) polyunsaturated to saturated fat ratio 6.) trans fat 7.) alcohol 8.) long-term multivitamin use 9.) white: red meat ratio ^f^ AHA 2020 dietary pattern was comprised of eight components: 1.) fruits & vegetables 2.) fish 3.) fiber-rich whole grains 4.) nuts, legumes & seeds 5.) sodium 6.) sugar-sweetened beverages 7.) processed meats 8.) saturated fat ^g^ Multivariate model: adjusted for age (years), sex (male vs. female), race (Caucasian vs. non-Caucasian), enrollment site (4 clinics), education (< high school, =high school, >high school), annual income (<$25 000, $25 000-$49 999, >$50 000), kcal physical activity (quintiles), walking pace (<2 mph, 2-3mph, >3mph), smoking (never, former, current), alcohol intake (none, <1 drink/wk, 1-2 drink/wk, ≥3 drink/wk) ^h^ + Mediator adjusted: Multivariate model + additional adjustment for potential mediators, including body mass index (kg/m^2^), prevalent treated hypertension (yes vs. no), prevalent diabetes mellitus (yes vs. no)

**SUPPLEMENTARY TABLE 7.** Relative risk of incident HF by lifestyle risk factors in older US adults without prevalent CHD (n=3734)^a^

| HR (95% CI) .  % of total Person-years Cases Multivariate Multivariate + lifestyle Multivariate + mediator  participants of follow-up Model^b^ Model^c^  Model^d^ | | | | | | |
| --- | --- | --- | --- | --- | --- | --- |
| Healthy diet pattern^e^  Q1  Q2  Q3  Q4  Q5 | 19.9  20.5  21.8  20.4  17.4 | 8390  8698  9051  9184  9553 | 209  253  209  210  175 | 1.0 (ref)  1.11 (0.93-1.34)  1.05 (0.87-1.28)  1.02 (0.84-1.25)  0.97 (0.79-.1.19) | 1.0 (ref)  1.25 (1.04-1.51)  1.04 (0.86-1.27)  1.04 (0.85-1.27)  0.92 (0.74-1.15) | 1.0 (ref)  1.21 (1.00-1.45)  1.00 (0.83-1.23)  1.01 (0.83-1.23)  0.89 (0.71-1.10) |
| Walking pace  < 2 mph  2-3 mph  > 3 mph | 27.2  43.4  29.4 | 10679  19296  14901 | 331  452  273 | 1.0 (ref)  0.79 (0.68-0.91)  0.65 (0.55-0.77) | 1.0 (ref)  0.83 (0.71-0.97)  0.71 (0.60-0.85) | 1.0 (ref)  0.86 (0.74-1.00)  0.78 (0.65-0.93) |
|  |  |  |  |  |  |  |
| Leisure activity, kcal/wk |  |  |  |  |  |  |
| Q1 (0-330)  Q2 (331-844)  Q3 (845-1530)  Q4 (1531-2945)  Q5 (≥ 2946) | 19.9  20.0  20.2  19.8  20.1 | 8141  8515  9255  9225  9571 | 231  235  201  189  196 | 1.0 (ref)  1.01 (0.84-1.21)  0.76 (0.62-0.91)  0.73 (0.60-0.88)  0.73 (0.60-0.88) | 1.0 (ref)  1.03 (0.85-1.26)  0.77 (0.62-0.95)  0.75 (0.60-0.93)  0.76 (0.61-0.96) | 1.0 (ref)  1.05 (0.86-1.28)  0.81 (0.65-1.00)  0.77 (0.61-0.96)  0.83 (0.66-1.05) |
| Smoking  Never  Former  Current  Alcohol^f^  None  <1 drink/wk  1-3 drink/wk  ≥ 3 drink/wk | 47.5  40.5  12.0  47.6  24.2  10.6  17.6 | 22305  17725  4847  20469  11288  5136  7983 | 480  457  119  552  242  105  157 | 0.68 (0.55-0.83)  0.85 (0.70-1.05)  1.0 (ref)  1.0 (ref)  0.80 (0.70-0.92)  0.71 (0.58-0.85)  0.71 (0.61-0.84) | 0.62 (0.50-0.76)  0.83 (0.67-1.02)  1.0 (ref)  1.0 (ref)  0.78 (0.69-0.90)  0.68 (0.56-0.83)  0.70 (0.60-0.84) | 0.55 (0.44-0.68)  0.72 (0.58-0.89)  1.0 (ref)  1.0 (ref)  0.82 (0.71-0.94)  0.74 (0.61-0.89)  0.74 (0.63-0.88) |
|  |  |  |  |  |  |  |
| Body mass index  < 22.0  22.0-24.9  25.0-29.9  ≥ 30.0 | 13.9  24.1  42.6  19.4 | 6183  10884  19095  8713 | 126  223  441  226 | 0.58 (0.46-0.72)  0.60 (0.50-0.72)  0.67 (0.58-0.79)  1.0 (ref) | 0.57 (0.46-0.72)  0.63 (0.52-0.77)  0.68 (0.58-0.80)  1.0 (ref) | 0.66 (0.52-0.83)  0.70 (0.58-0.85)  0.72 (0.61-0.85)  1.0 (ref) |
|  |  |  |  |  |  |  |
| Waist circumference^g^  < 88cm women, < 92cm men  ≥ 88cm women, ≥ 92cm men | 36.7  63.3 | 17102  27774 | 334  722 | 0.76 (0.66-0.86)  1.0 (ref) | 0.78 (0.68-0.89)  1.0 (ref) | 0.83 (0.72-0.95)  1.0 (ref) |

^a^ Data are HR (95% CI) based on cumulatively updated exposures ^b^ Multivariate model was adjusted for age (years), sex (male vs. female), race (Caucasian vs. non-Caucasian), enrollment site (4 clinics), education (< high school, =high school, >high school), annual income (<$25 000, $25 000-$49 999, >$50 000) ^c^ Multivariate + lifestyle model was mutually adjusted for other lifestyle factors in the table [categorization: healthy diet pattern (quintiles), leisure activity, kcal/wk (quintiles), walking pace (<2 mph, 2-3mph, >3mph), smoking (never, former, current), alcohol intake (none, <1 drink/wk, 1-3 drink/wk, ≥3 drink/wk), body mass index (<22, 22-24.9, 25-29.9, ≥30kg/m^2^] ^d^ Multivariate + mediator model was adjusted for all covariates in the multivariate + lifestyle model, with additional adjustment for potential mediators including body mass index (kg/m^2^), prevalent treated hypertension (yes vs. no), prevalent diabetes mellitus (yes vs. no) ^e^ The Biologic pattern was used for the healthy diet score. The components of dietary pattern included fruits, vegetables, whole grains, fish/seafood, polyunsaturated to saturated fat ratio, nuts/seeds, red and processed meats, sugar-sweetened beverages, trans fat, and sodium. Results were similar using DASH, AHEI, or AHA 2020 instead of the Biologic pattern ^f^ Alcohol intake was modest; <10% of adults consumed > 2 drinks/week ^g^ The low-risk classifications were based on the observed distribution of risk factors within the CHS cohort and current recommendations, when available; for instance, low risk for waist circumference was defined as less than 88 cm in women or 92 cm in men based on Adult Treatment Panel III criteria

**SUPPLEMENTARY TABLE 8.** Relative risk of incident HF by quintiles of dietary components in older US adults without prevalent CHD (n=3734)^a^

| . Quintiles of intake .  1 2 3 4 5 *P*(trend)^b^ | | | | | | |
| --- | --- | --- | --- | --- | --- | --- |
| Fruits  Multivariate^c^  + Mediator adjusted^d^ | 1.0 (ref)  1.0 (ref) | 0.80 (0.65-0.96)  0.79 (0.64-0.97) | 1.02 (0.85-1.23)  0.97 (0.86-1.20) | 1.11 (0.92-1.33)  1.04 (0.87-1.26) | 0.92 (0.75-1.12)  0.89 (0.73-1.08) | 0.56  0.90 |
|  |  |  |  |  |  |  |
| Vegetables  Multivariate  + Mediator adjusted | 1.0 (ref)  1.0 (ref) | 1.07 (0.88-1.30)  1.02 (0.85-1.25) | 1.21 (0.99-1.46)  1.16 (0.96-1.41) | 1.16 (0.95-1.42)  1.12 (0.92-1.37) | 1.23 (1.00-1.51)  1.19 (0.97-1.46) | 0.16  0.23 |
|  |  |  |  |  |  |  |
| Whole grains |  |  |  |  |  |  |
| Multivariate  + Mediator adjusted | 1.0 (ref)  1.0 (ref) | 0.88 (0.73-1.07)  0.84 (0.69-1.02) | 1.11 (0.92-1.34)  1.11 (0.92-1.33) | 1.01 (0.84-1.23)  1.00 (0.82-1.21) | 0.97 (0.80-1.17)  0.96 (0.79-1.17) | 0.75  0.72 |
|  |  |  |  |  |  |  |
| Fish |  |  |  |  |  |  |
| Multivariate  + Mediator adjusted | 1.0 (ref)  1.0 (ref) | 1.06 (0.88-1.28)  1.04 (0.86-1.25) | 1.09 (0.91-1.32)  1.09 (0.91-1.31) | 1.04 (0.85-1.26)  1.00 (0.83-1.22) | 1.02 (0.83-1.25)  0.98 (0.80-1.20) | 0.88  0.55 |
|  |  |  |  |  |  |  |
| PUFA/SF ratio^e^ |  |  |  |  |  |  |
| Multivariate  + Mediator adjusted | 1.0 (ref)  1.0 (ref) | 1.09 (0.90-1.31)  1.07 (0.89-1.29) | 1.08 (0.90-1.30)  1.06 (0.88-1.28) | 0.89 (0.73-1.07)  0.90 (0.74-1.09) | 0.92 (0.76-1.12)  0.93 (0.77-1.14) | 0.11  0.18 |
|  |  |  |  |  |  |  |
| Nuts/seeds  Multivariate  + Mediator adjusted | 1.0 (ref)  1.0 (ref) | 0.94 (0.78-1.13)  0.92 (0.76-1.10) | 0.82 (0.69-1.00)  0.81 (0.66-0.98) | 0.87 (0.72-1.05)  0.87 (0.72-1.05) | 0.86 (0.71-1.04)  0.85 (0.70-1.03) | 0.40  0.42 |
|  |  |  |  |  |  |  |
| Red & processed meat^g^  Multivariate  + Mediator adjusted | 1.0 (ref)  1.0 (ref) | 1.09 (0.80-1.32)  1.01 (0.95-1.27) | 1.04 (0.86-1.27)  1.11 (1.84-1.47) | 1.09 (0.90-1.33)  1.12 (0.85-1.48) | 1.16 (0.95-1.41)  1.21 (0.92-1.60) | 0.19  0.19 |
| Red meat (unprocessed)^f^  Multivariate  + Mediator adjusted | 1.0 (ref)  1.0 (ref) | 0.83 (0.68-1.00)  0.81 (0.66-0.99) | 0.96 (0.80-1.16)  0.94 (0.78-1.13) | 0.96 (0.79-1.16)  0.94 (0.77-1.13) | 0.86 (0.80-1.16)  0.96 (0.79-1.19) | 0.40  0.36 |
| Processed meat^g^  Multivariate  + Mediator adjusted  Sugar-sweetened beverages  Multivariate  + Mediator adjusted | 1.0 (ref)  1.0 (ref)  1.0 (ref)  1.0 (ref) | 1.07 (0.91-1.26)  1.07 (0.91-1.27)  0.93 (0.78-1.10)  0.92 (0.77-1.09) | 1.19 (0.85-1.66)  1.20 (0.86-1.67)  0.91 (0.77-1.09)  0.94 (0.79-1.12) | 1.09 (0.77-1.54)  1.12 (0.79-1.58)  0.95 (0.80-1.13)  0.96 (0.81-1.14) | 1.16 (0.82-1.65)  1.21 (0.99-1.79)  0.96 (0.81-1.14)  0.97 (0.81-1.15) | 0.34  0.17  0.28  0.32 |
| Trans fat  Multivariate  + Mediator adjusted | 1.0 (ref)  1.0 (ref) | 1.12 (0.92-1.36)  1.12 (0.92-1.37) | 1.10 (0.90-1.35)  1.10 (0.90-1.35) | 1.26 (1.03-1.53)  1.26 (1.03-1.53) | 0.99 (0.81-1.22)  1.00 (0.81-1.23) | 0.96  0.96 |
| Sodium  Multivariate  + Mediator adjusted | 1.0 (ref)  1.0 (ref) | 1.11 (0.93-1.32)  1.14 (0.93-1.29) | 1.18 (0.99-1.40)  1.21 (0.99-1.48) | 1.26 (1.06-1.49)  1.30 (1.06-1.59) | 1.19 (1.00-1.41)  1.25 (1.03-1.53) | **0.014**  **0.009** |

^a^ Data are HR (95% CI) based on cumulatively updated intake. The foods were components of the Biologic dietary pattern ^b^ Linear trend was tested by assigning the median value to participants in each quintile and entering this into the model as a continuous variable ^c^ Multivariate model: adjusted for age (years), sex (male vs. female), race (Caucasian vs. non-Caucasian), enrollment site (4 clinics), education (< high school, =high school, >high school), annual income (<$25 000, $25 000-$49 999, >$50 000), total kcal expended (quintiles), walking pace (<2 mph, 2-3mph, >3mph), smoking (never, former, current), alcohol intake (none, <1 drink/wk, 1-2 drink/wk, ≥3 drink/wk) ^d^ + Mediator adjusted: Multivariate model + additional adjustment for potential mediators, including body mass index (kg/m^2^), prevalent treated hypertension (yes vs. no), prevalent diabetes mellitus (yes vs. no) ^e^ Polyunsaturated fat to saturated fat ratio ^f^ Red meat (unprocessed) was defined as beef steaks/roasts, beef stew, and pork chops/roasts ^g^ Processed meat was defined as hot dogs, ham, lunch meat, bacon, and sausage

**SUPPLEMENTARY TABLE 9.** Hazard ratios (95% CI) for incident heart failure by quintiles of diet-quality scores in older US adults in those with self-assessed excellent, very good, or good health (n=3395)^a^

| .Quintiles of diet-quality scores .  1 2 3 4 5 *P*(trend)^b^ | | | | | | |
| --- | --- | --- | --- | --- | --- | --- |
| Biologic^c^  Cases/person-years  Multivariate^g^  + Mediator adjusted^h^ | 187/7371  1.0 (ref)  1.0 (ref) | 281/7673  1.17 (0.96-1.43)  1.16 (0.95-1.41) | 208/8678  1.08 (0.88-1.32)  1.05 (0.86-1.29) | 207/8676  1.11 (0.90-1.36)  1.06 (0.86-1.31) | 178/9103  1.01 (0.81-1.26)  0.96 (0.77-1.20) | 0.89  0.53 |
| DASH^d^  Cases/person-years  Multivariate  + Mediator adjusted | 204/7880  1.0 (ref)  1.0 (ref) | 226/9457  1.02 (0.84-1.23)  1.01 (0.84-1.22) | 198/8425  1.06 (0.87-1.30)  1.01 (0.83-1.23) | 181/7715  1.11 (0.91-1.36)  1.08 (0.88-1.33) | 182/8024  1.09 (0.88-1.34)  1.03 (0.93-1.27) | 0.54  0.71 |
| AHEI^e^  Cases/person-years  Multivariate  + Mediator adjusted | 185/6863  1.0 (ref)  1.0 (ref) | 206/7497  1.12 (0.92-1.37)  1.09 (0.89-1.33) | 227/8978  1.10 (0.90-1.35)  1.06 (0.87-1.30) | 197/8393  1.07 (0.87-1.33)  1.04 (0.84-1.29) | 176/9769  0.93 (0.74-1.17)  0.89 (0.71-1.12) | 0.44  0.27 |
| AHA 2020^f^  Cases/person-years  Multivariate  + Mediator adjusted | 197/7338  1.0 (ref)  1.0 (ref) | 193/8248  0.92 (0.75-1.12)  0.95 (0.77-1.16) | 222/8312  1.10 (0.90-1.34)  1.05 (0.87-1.29) | 197/8960  1.07 (0.87-1.31)  1.03 (0.84-1.27) | 182/8644  0.96 (0.78-1.19)  0.93 (0.75-1.14) | 0.79  0.76 |

^a^ Data are HR (95% CI) based on cumulatively averaged Biologic score, Dietary Approaches to Stop Hypertension (DASH) score, the Alternative Healthy Eating Index (AHEI) score, and the American Heart Association dietary 2020 goals (AHA 2020) score ^b^ Linear trend was tested by assigning the median value to participants in each quintile and entering this into the model as a continuous variable ^c^ Biologic dietary pattern was comprised of ten components: 1.) fruits 2.) vegetables 3.) whole grains 4.) fish 5.) polyunsaturated to saturated fat ratio 6.) nuts/seeds 7.) red & processed meats 8.) sugar-sweetened beverages 9.) trans fat 10.) sodium. For scoring of dietary patterns, see **Supplementary Table 1**. ^d^ DASH dietary pattern was comprised of eight components: 1.) low-fat dairy 2.) fruits 3.) vegetables 4.) nuts & legumes 5.) whole grains 6.) red & processed meats 7.) sugar-sweetened beverages 8.) sodium ^e^ AHEI dietary pattern was comprised of nine components: 1.) fruits 2.) vegetables 3.) nuts & soy protein 4.) cereal fiber 5.) polyunsaturated to saturated fat ratio 6.) trans fat 7.) alcohol 8.) long-term multivitamin use 9.) white: red meat ratio ^f^ AHA 2020 dietary pattern was comprised of eight components: 1.) fruits & vegetables 2.) fish 3.) fiber-rich whole grains 4.) nuts, legumes & seeds 5.) sodium 6.) sugar-sweetened beverages 7.) processed meats 8.) saturated fat ^g^ Multivariate model: adjusted for age (years), sex (male vs. female), race (Caucasian vs. non-Caucasian), enrollment site (4 clinics), education (< high school, =high school, >high school), annual income (<$25 000, $25 000-$49 999, >$50 000), kcal physical activity (quintiles), walking pace (<2 mph, 2-3mph, >3mph), smoking (never, former, current), alcohol intake (none, <1 drink/wk, 1-2 drink/wk, ≥3 drink/wk) ^h^ + Mediator adjusted: Multivariate model + additional adjustment for potential mediators, including body mass index (kg/m^2^), prevalent treated hypertension (yes vs. no), prevalent diabetes mellitus (yes vs. no), prevalent coronary heart disease (yes vs. no)

**SUPPLEMENTARY TABLE 10.** Relative risk of incident heart failure by lifestyle risk factors in older US adults by self-perceived health status^a^

|  | HR (95% CI) .  ..All participants No prevalent CHD Fair or better health^b^  Good or better health^c^ …….n=4490 n=3734 n=3275 n=2813  …….n=1390 n=1056 n=973 n=764 | | | | | | | | | | |  |  |
| --- | --- | --- | --- | --- | --- | --- | --- | --- | --- | --- | --- | --- | --- |
| Healthy diet pattern^d^  Q1  Q2  Q3  Q4  Q5 | | 1.0 (ref)  1.19 (1.00-1.40)  1.05 (0.88-1.25)  1.08 (0.91-1.29)  0.99 (0.82-1.19) | | 1.0 (ref)  1.21 (1.00-1.45)  1.00 (0.83-1.23)  1.01 (0.83-1.23)  0.89 (0.71-1.10) | | | 1.0 (ref)  1.03 (0.84-1.25)  0.95 (0.77-1.16)  1.16 (0.94-1.43)  1.07 (0.86-1.34) | | 1.0 (ref)  1.22 (0.97-1.52)  0.98 (0.77-1.24)  1.11 (0.88-1.44)  1.01 (0.79-1.38) | |  |  |  |
| Walking pace  < 2 mph  2-3 mph  > 3 mph | | 1.0 (ref)  0.87 (0.76-0.99)  0.79 (0.68-0.92) | | 1.0 (ref)  0.86 (0.74-1.00)  0.78 (0.65-0.93) | | | 1.0 (ref)  0.97 (0.82-1.14)  0.85 (0.71-1.03) | | 1.0 (ref)  1.02 (0.84-1.23)  0.94 (0.76-1.16) | |  |  |  |
|  | |  |  | |  |  | |  | |  | | |  |
| Leisure activity, kcal/wk | |  |  | |  |  | |  | |  | | |  |
| Q1 (0-330)  Q2 (331-844)  Q3 (845-1530)  Q4 (1531-2945)  Q5 (≥ 2946) | | 1.0 (ref)  1.00 (0.85-1.18)  0.78 (0.65-0.92)  0.81 (0.68-0.96)  0.82 (0.69-0.98) | | 1.0 (ref)  1.05 (0.86-1.28)  0.81 (0.65-1.00)  0.77 (0.61-0.96)  0.83 (0.66-1.05) | | | 1.0 (ref)  1.08 (0.88-1.34)  0.79 (0.64-0.98)  0.82 (0.66-1.02)  0.88 (0.71-1.09) | | 1.0 (ref)  1.22 (0.97-1.52)  0.98 (0.77-1.24)  1.11 (0.88-1.44)  1.01 (0.79-1.30) | |  |  |  |
| Smoking  Never  Former  Current  Alcohol^e^  None  <1 drink/wk  1-3 drink/wk  ≥ 3 drink/wk | | 0.60 (0.49-0.72)  0.72 (0.60-0.87)  1.0 (ref)  1.0 (ref)  0.82 (0.71-0.94)  0.74 (0.61-0.89)  0.74 (0.63-0.88) | | 0.55 (0.44-0.68)  0.72 (0.58-0.89)  1.0 (ref)  1.0 (ref)  0.82 (0.71-0.94)  0.74 (0.61-0.89)  0.74 (0.63-0.88) | | | 0.54 (0.43-0.68)  0.73 (0.58-0.91)  1.0 (ref)  1.0 (ref)  0.86 (0.73-1.01)  0.75 (0.60-0.94)  0.70 (0.57-0.85) | | 0.65 (0.50-0.84)  0.86 (0.66-1.12)  1.0 (ref)  1.0 (ref)  0.99 (0.82-1.19)  0.84 (0.66-1.07)  0.77 (0.62-0.96) | |  |  |  |
|  | |  |  | |  |  | |  | |  | | |  |
| Body mass index  < 22.0  22.0-24.9  25.0-29.9  ≥ 30.0 | | 0.73 (0.59-0.88)  0.71 (0.60-0.84)  0.80 (0.69-0.92)  1.0 (ref) | | 0.66 (0.52-0.83)  0.70 (0.58-0.85)  0.72 (0.61-0.85)  1.0 (ref) | | | 0.66 (0.52-0.84)  0.75 (0.61-0.91)  0.75 (0.63-0.89)  1.0 (ref) | | 0.60 (0.46-0.80)  0.71 (0.57-0.89)  0.75 (0.61-0.91)  1.0 (ref) | |  |  |  |
|  | |  |  | |  |  | |  | |  | | |  |
| Waist circumference^f^  < 88cm women, < 92cm men  ≥ 88cm women, ≥ 92cm men | | 0.86 (0.77-0.97)  1.0 (ref) | | 0.83 (0.72-0.95)  1.0 (ref) | | | 0.84 (0.75-0.96)  1.0 (ref) | | 0.82 (0.70-0.96)  1.0 (ref) | |  |  |  |

^a^ Data are HR (95% CI) based on cumulatively updated exposures. Multivariate models were adjusted for age (years), sex (male vs. female), race (Caucasian vs. non-Caucasian), enrollment site (4 clinics), education (< high school, =high school, >high school), annual income (<$25 000, $25 000-$49 999, >$50 000), mutually adjusted for other lifestyle factors in the table [categorization: healthy diet pattern (quintiles), leisure activity, kcal/wk (quintiles), walking pace (<2 mph, 2-3mph, >3mph), smoking (never, former, current), alcohol intake (none, <1 drink/wk, 1-3 drink/wk, ≥3 drink/wk), body mass index (<22, 22-24.9, 25-29.9, ≥30kg/m2], and additionally adjusted for potential mediators including body mass index (kg/m2), hypertension (yes vs. no), and diabetes mellitus (yes vs. no) ^b^ Fair or better health excluded those with prevalent CHD, those with self-described poor health, and those with difficulty with activities of daily living ^c^ Good or better health excluded those with prevalent CHD, those with self-described fair or poor health, and those with difficulty with activities of daily living ^d^ The Biologic pattern was used for the healthy diet score. The components of dietary pattern included fruits, vegetables, whole grains, fish/seafood, polyunsaturated to saturated fat ratio, nuts/seeds, red and processed meats, sugar-sweetened beverages, trans fat, and sodium. Results were similar using DASH, AHEI, or AHA 2020 instead of the Biologic pattern ^e^ Alcohol intake was modest; <10% of adults consumed > 2 drinks/week ^f^ The low-risk classifications were based on the observed distribution of risk factors within the CHS cohort and current recommendations, when available; for instance, low risk for waist circumference was defined as less than 88 cm in women or 92 cm in men based on Adult Treatment Panel III criteria

**SUPPLEMENTARY TABLE 11.** Relative risk of incident HF by lifestyle factors and adiposity in US older adults, with and without adjustment for NT-proBNP (n=4490)^a^

| _____ HR (95% CI) _________ .  % of total Person-years Heart failure Multivariate + Multivariate +  participants of follow-up cases Lifestyle Model^b^ Lifestyle + NT-proBNP^c^ | | | | | | |  |
| --- | --- | --- | --- | --- | --- | --- | --- |
| Healthy diet pattern^d^  Lower 2 quintiles  Upper 3 quintiles | 36.1  63.9 | 19605  32244 | 583  797 | 1.0 (ref)  0.98 (0.87-1.09) | 1.0 (ref)  1.00 (0.88-1.14) |  | |
| Walking pace  < 2mph  ≥ 2mph | 28.6  71.4 | 12770  39079 | 454  926 | 1.0 (ref)  0.80 (0.71-0.90) | 1.0 (ref)  0.82 (0.71-0.94) |  | |
|  |  |  |  |  |  |  | |
| Leisure activity, kcal/wk^e^  < 845  ≥ 845 | 41.2  58.8 | 19775  32074 | 624  756 | 1.0 (ref)  0.78 (0.69-0.87) | 1.0 (ref)  0.70 (0.62-0.80) |  | |
|  |  |  |  |  |  |  | |
| Smoking  Current  Never or former | 11.6  88.4 | 5452  46397 | 149  1231 | 1.0 (ref)  0.71 (0.59-0.88) | 1.0 (ref)  0.70 (0.57-0.87) |  | |
|  |  |  |  |  |  |  | |
| Alcohol intake^f^  < 1 drink/wk  ≥ 1 drink/wk | 72.3  27.7 | 36803  15045 | 1040  340 | 1.0 (ref)  0.77 (0.67-0.88) | 1.0 (ref)  0.79 (0.68-0.92) |  | |
|  |  |  |  |  |  |  | |
| Body mass index  ≥ 30.0  < 30.0 | 19.2  80.8 | 10050  41799 | 336  1044 | 1.0 (ref)  0.70 (0.61-0.80) | 1.0 (ref)  0.74 (0.65-0.85) |  | |

^a^ Data are HR (95% CI) based on cumulatively updated exposures ^b^ Multivariate + lifestyle model was mutually adjusted for other lifestyle factors in the table [categorization: healthy diet pattern (quintiles), leisure activity, kcal/wk (quintiles), walking pace (<2 mph, 2-3mph, >3mph), smoking (never, former, current), alcohol intake (none, <1 drink/wk, 1-3 drink/wk, ≥3 drink/wk), body mass index (kg/m^2^)] ^c^ Model additionally adjusted for baseline NT-proBNP ^d^ The Biologic pattern was used for the healthy diet score. The components of dietary pattern included fruits, vegetables, whole grains, fish/seafood, polyunsaturated to saturated fat ratio, nuts/seeds, red and processed meats, sugar-sweetened beverages, trans fat, and sodium. Results were similar using DASH, AHEI, or AHA 2020 instead of the Biologic pattern ^e^ kcal cut-off approximates amount of energy expended by adhering to CDC physical activity recommendations for older adults to achieve ‘important health benefits’ ^f^ Alcohol intake was modest; <10% of adults consumed > 2 drinks/week
